# L-Cysteine-Modified Transfersomes for Enhanced Epidermal Delivery of Podophyllotoxin

**DOI:** 10.3390/molecules28155712

**Published:** 2023-07-28

**Authors:** Jiangxiu Niu, Ming Yuan, Jingjing Chen, Liye Wang, Yueheng Qi, Kaiyue Bai, Yanli Fan, Panpan Gao

**Affiliations:** College of Food and Drug, Luoyang Normal University, Luoyang 471934, China; niujiangxiu@lynu.edu (J.N.); yuanming@lynu.edu.cn (M.Y.); 18338462749@163.com (J.C.); bky31415926@163.com (K.B.); fyl2023@lynu.edu.cn (Y.F.);

**Keywords:** transfersome, drug delivery, L-cysteine, epidermal delivery, podophyllotoxin

## Abstract

The purpose of this study was to evaluate L-cysteine-modified transfersomes as the topical carrier for enhanced epidermal delivery of podophyllotoxin (POD). L-cysteine-deoxycholic acid (LC-DCA) conjugate was synthesized via an amidation reaction. POD-loaded L-cysteine-modified transfersomes (POD-LCTs) were prepared via a thin membrane dispersion method and characterized for their particle size, zeta potential, morphology, X-ray diffraction (XRD), Fourier transform infrared spectroscopy (FTIR) and in vitro release. Subsequently, in vitro skin permeation and retention, fluorescence distribution in the skin, hematoxylin–eosin staining and in vivo skin irritation were studied. The POD-LCTs formed spherical shapes with a particle size of 172.5 ± 67.2 nm and a zeta potential of −31.3 ± 6.7 mV. Compared with the POD-Ts, the POD-LCTs provided significantly lower drug penetration through the porcine ear skin and significantly increased the skin retention (*p* < 0.05). Meaningfully, unlike the extensive distribution of the POD-loaded transfersomes (POD-Ts) throughout the skin tissue, the POD-LCTs were mainly located in the epidermis. Moreover, the POD-LCTs did not induce skin irritation. Therefore, the POD-LCTs provided an enhanced epidermal delivery and might be a promising carrier for the topical delivery of POD.

## 1. Introduction

Podophyllotoxin (POD) can inhibit the division and proliferation of epithelial cells infected by human papillomavirus (HPV) in active epidermis, and it is usually used as the first-line drug in the treatment of genital warts [1]. Since the systemic absorption of POD will cause serious side effects, it is necessary to improve the drug accumulation in the skin and reduce the systemic absorption through topical administration [2]. However, POD is usually used clinically in the form of common topical preparations such as a tincture or cream, which will lead to systemic toxic side effects and severe skin irritation [3]. Therefore, it is very important and meaningful for patients with genital warts to improve POD accumulation in the active epidermis and reduce its side effects during clinical use. In order to overcome the obstacles related to conventional preparations, new formulation is needed to deliver drugs to the focal site with reduced side effects and increased activity.

In recent years, the application of nanocarriers in drug delivery systems has attracted the attention of researchers because of their ability to effectively deliver drugs to lesions [4,5,6,7,8,9]. In this vein, the drug delivery strategy based on nanoparticles provides a promising and encouraging way to improve the efficacy of POD and reduce its side effects. Up to now, POD has been encapsulated by many types of nanoparticles, such as solid lipid nanoparticles [2], poly-D, L-lactide nanoparticles [10] and lipid bilayer nanoparticles [11]. The transfersome is composed of a phospholipid bilayer and edge activators (such as sodium cholic acid, sodium deoxycholic acid, span 80, Tween 80 and dipotassium glycyrrhizate) [12]. With the help of the edge activators, transfersome can squeeze into and penetrate the intercellular lipid channels in the stratum corneum, thus enhancing the skin penetration and deposition of drugs [13]. For the topical treatment of genital warts, drug delivery to the active epidermis is essential because HPV is present in the basal layer cells of the active epidermis [14]. However, transfersomes can pass through the stratum corneum and active epidermis and enter the blood circulation for the whole body, leading to system side effects. Therefore, how to retain transfersomes in the active epidermis after penetrating the stratum corneum is an important problem to be solved.

In recent decades, nanoparticles with bioadhesive properties have attracted much attention as a promising tool for improving the effectiveness of drug delivery systems [15]. These nanoparticles can adhere to local action sites through hydrogen bonding or non-covalent electrostatic interactions. Another kind of bioadhesive nanoparticles, such as sulfhydryl modified nanoparticles, has the ability to bind to specific sites and achieve local targeting. They exhibit free sulfhydryl groups that lead to covalent bonding to the cysteine-rich substructural regions of the tissue, thereby increasing the residence time at the action site. Sulfhydryl-modified nanoparticles have been widely used to improve intestinal absorption of oral drugs [16], enhance mucosal delivery of drugs [17], prolong the residence time of nanoparticles at cancer sites [18] and increase the retention of micelles in human corneal epithelium (HCEC) cells [19]. L-cysteine is a well-known nonessential amino acid containing sulfhydryl groups that can be oxidized to cysteine, which has a variety of pharmacological effects, including antioxidant and anti-inflammatory activities [20]. Skin keratin is the main structural protein of epidermal cells. The structure of skin keratin is rich in cysteine, and nanoparticles modified with L-cysteine can improve its epidermal retention ability through covalently interacting with keratin [21].

**Figure 1:**
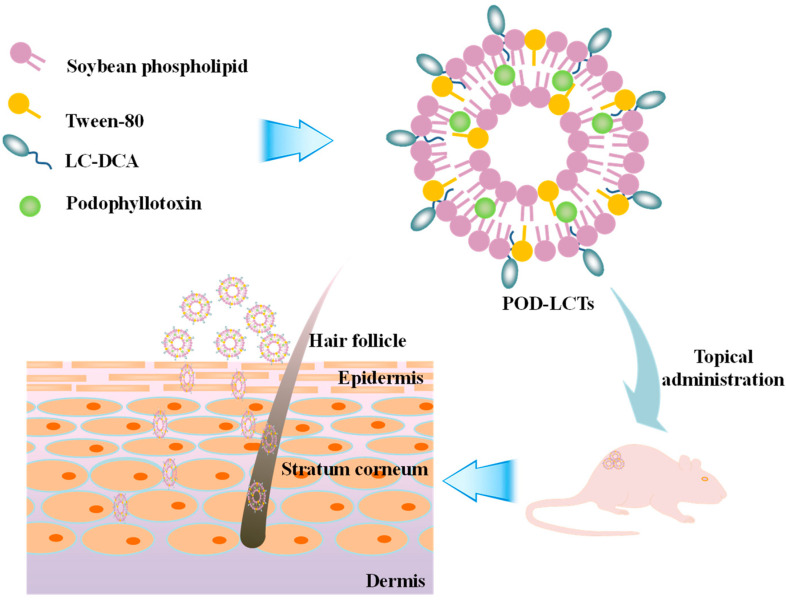
Schematic illustration of formulation and application of POD-LCTs for enhanced epidermal delivery of POD.

In the present study, L-cysteine-modified transfersomes were developed for enhanced epidermal delivery of POD (Figure 1). L-cysteine-deoxycholic acid (LC-DCA) conjugate was synthesized via an amidation reaction. POD-loaded L-cysteine-modified transfersomes (POD-LCTs) were prepared through a thin membrane dispersion method. The physicochemical properties of the formulation were characterized in terms of the encapsulation efficiency (EE), particle size, zeta potential, morphology, X-ray powder diffraction (XRD), Fourier transform infrared spectroscopy (FTIR) and elasticity. The in vitro release of POD from the developed formulation was studied with a Franz diffusion cell. Furthermore, the in vitro skin permeation and deposition of the formulation and the fluorescence distribution of the developed formulation in the skin were focused upon. In addition, the effects of the formulation on the microstructure of the skin were also studied. Finally, in vivo skin irritation tests were conducted in mice to evaluate the biocompatibility and safety of the formulation.

## 2. Results and Discussion

### 2.1. Synthesis and Characterization of the LC-DCA Conjugate

**Figure 2:**
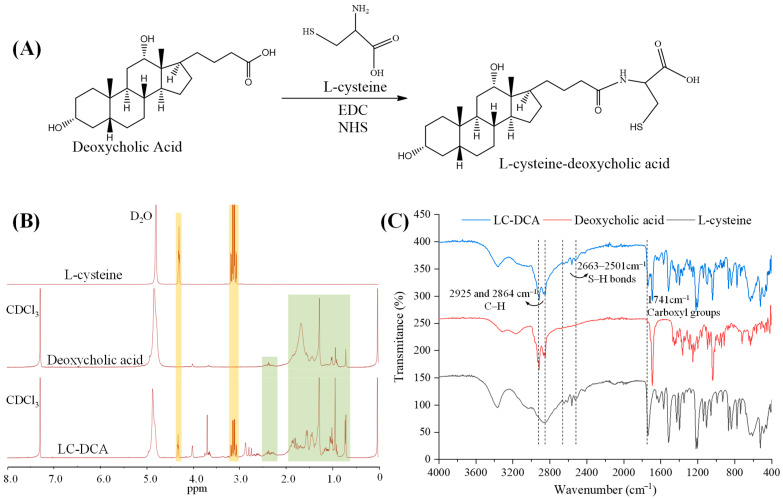
(**A**) Synthetic scheme of LC-DCA conjugate. (**B**) ^1^H NMR spectra of L-cysteine, deoxycholic acid and LC-DCA conjugate (The orange area represents the peaks belonging to L-cysteine and the green area represents the peaks belonging to deoxycholic acid). (**C**) FTIR of L-cysteine, deoxycholic acid and LC-DCA conjugate.

The LC-DCA conjugate was synthesized by mild catalytic chemical reaction (Figure 2A). EDC and NHS were used as catalysts to react with the carboxyl group of DCA to form a highly active unstable ester intermediate, which then reacted with the primary amine of LC to form an amide bond and finally synthesized the conjugate of LC-DCA. The reaction was carried out in a PBS buffer/methanol mixed solvent due to the low solubility of DCA in aqueous solution. The chemical structure of LC-DCA was identified by ^1^H NMR (Figure 2B). It can be seen from the spectrum of LC-DCA that peaks which appeared in the range of 3.05–3.27 ppm and 4.20–4.35 ppm were ascribed to the –CH_2_– and –CH– signals of LC [21], and peaks which appeared in the range of 0.64–2.50 ppm were assigned to the –CH_3_– and –CH_2_– protons of DCA [22], which indicated the successful introduction of L-cysteine into DCA.

FTIR analysis was also carried out to assist in confirming the synthesis of the LC-DCA conjugate (Figure 2C). In the FTIR spectra of DCA and the synthetic product, the signals present at 2925 and 2864 cm^−1^ were attributed to the C–H stretching vibration of the methylene of DCA. These signals were also described by Liang et al. when evaluating the FTIR spectra of DCA [22]. Regarding the LC, the resulting FTIR spectra showed several characteristic peaks in the region of 2663–2501 cm^−1^, corresponding to the stretch vibration of the S-H bonds, and a peak at 1741 cm^−1^ was also recorded, which was attributed to the stretching vibration of the carboxyl groups [23]. The same signal was identified in the LC-DCA conjugate. Thus, according to ^1^H NMR and FTIR analysis, it was possible to infer that the synthetic product corresponded to the LC-DCA conjugate, which was used in subsequent L-Cysteine-modified transfersome preparation. Macroscopic and sensory evaluation of the lyophilized conjugate revealed that it was a white-to-yellowish, tasteless and amorphous powder.

Since sulfhydryl conjugates could form disulfide bonds with keratin in the skin through free sulfhydryl groups to produce adhesion and retention, the amount of free sulfhydryl groups in the conjugate is very important. The amount of free sulfhydryl groups was measured using Ellman’s test, and the result showed about 1.81 × 10^−3^ μmol of sulfhydryl groups in 1 g LC-DCA conjugate, demonstrating the efficiency of the conjugate synthesis. At the same time, the high amount of free sulfhydryl groups in the conjugate would be beneficial to the topical skin retention of modified nanocarriers.

### 2.2. Preparation of POD-LCTs

Ideally, the more LC-DCA contained in the formulation, the better the targeting effect on the epidermis. However, excessive addition of LC-DCA might lead to system instability, with abnormal changes in particle size, zeta potential and EE. Therefore, POD-LCT formulations containing 20 mg, 40 mg, 60 mg and 80 mg LC-DCA were prepared and investigated. Table 1 shows that these characteristics varied with the amount of LC-DCA. When the amount of LC-DCA exceeded 60 mg, the particle size of the POD-LCTs increased from 172.5 nm to 192.0 nm, the particle size distribution became wider (the PDI value increased), and a bimodal phenomenon appeared. The decrease in the amount of POD encapsulated in the POD-LCTs also indicates that an amount of LC-DCA exceeding 60 mg might lead to instability in the system. Therefore, the optimal amount of LC-DCA was determined to be 60 mg in POD-LCT formulation.

### 2.3. Characterization of POD-LCTs

#### 2.3.1. Particle Size and Zeta Potential

**Figure 3:**
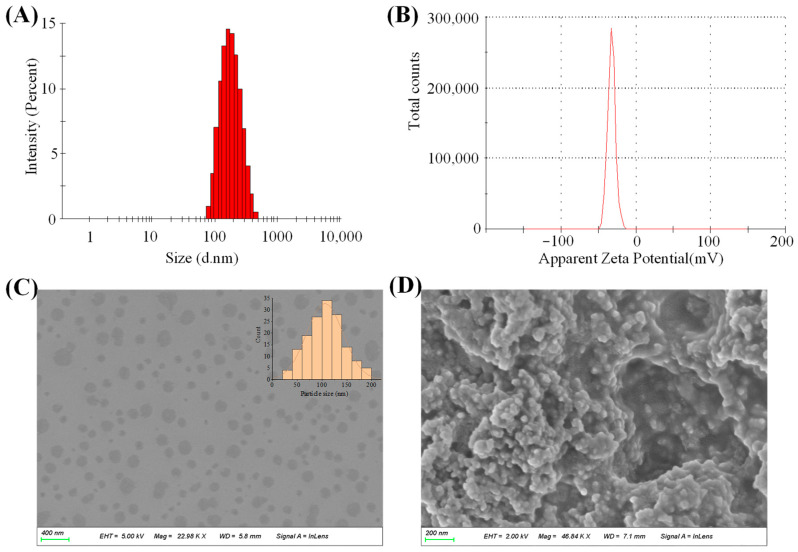
(**A**) Particle size distribution of POD-LCTs. (**B**) Zeta potential of POD-LCTs. (**C**) SEM image of POD-LCTs. (**D**) SEM image of lyophilized POD-LCTs.

The particle size, PDI and zeta potential of the POD-LCT formulation were 172.5 ± 67.2 nm, 0.14 ± 0.08 and −31.3 ± 6.7 mV (Figure 3A,B), respectively. The particle size of the POD-LCTs was within the expected range of 100–200 nm, which was considered to be conducive to the drug formulations to squeeze themselves between the pores of the stratum corneum and enter the active epidermis [24]. The PDI value of the POD-LCTs was 0.14, indicating that the size distribution of the POD-LCTs was narrow and the vesicle size was uniform. The zeta potential is an important physical parameter for predicting the stability of vesicles. Dispersed drug delivery systems with an absolute value of a zeta potential greater than 30 mV are considered stable [25]. The POD-LCTs showed negative zeta potentials, which might be due to the presence of carboxyl from cysteine and phosphate groups from phospholipids on the particle surface. It is well known that the skin may act as a negatively charged membrane [26]. The electrostatic interaction between the negatively charged skin surface and the optimized formulation might cause temporary channels in the skin structure, favoring the delivery and retention of the drug down to the deeper skin [27,28,29]. According to previous studies, negatively charged nanocarriers could improve the skin permeability and retention of drugs in transdermal delivery [30]. Therefore, the negative charge of the optimized formulation might affect the penetration of POD-LCTs through the skin.

#### 2.3.2. Morphology

SEM was used to observe the morphology of the POD-LCTs and confirm the formation of the vesicles. SEM images of the POD-LCT dispersion and lyophilized POD-LCTs are shown in Figure 3C,D. The morphology of the POD-LCT dispersion was spherical, the particle size distribution was uniform, and there was no aggregation phenomenon, indicating that the POD-LCTs formed single spherical vesicles. The morphology of the lyophilized POD-LCTs was loose spherical aggregates, probably due to the aggregation of vesicles during the freeze-drying process. The morphology of the lyophilized powder did not change, indicating that the POD-LCTs did not undergo structural changes after lyophilization. The size of the POD-LCTs decreased enormously following lyophilization when compared with the original POD-LCT dispersion. A size decrease was also observed in other drug-loaded nanocarriers due to lyophilization [31].

#### 2.3.3. XRD Study

**Figure 4:**
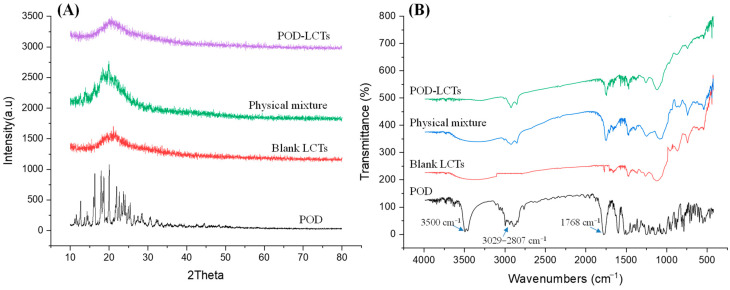
X-ray diffraction patterns (**A**) and FTIR spectra (**B**) of pure POD, blank LCTs, physical mixture and POD-LCTs.

The results of the XRD analysis are shown in Figure 4A. The XRD pattern of pure POD showed strong diffraction peaks of 12.68°, 16.33°, 17.95° and 20.01°, indicating that pure POD exists in a crystalline form [32]. The XRD pattern of the physical mixture shows that the typical peaks of the POD had a lower intensity due to the dominant role of the lipids used in the formulation. The diffraction patterns of the POD-LCTs and blank LCTs were similar, while there were significant differences between the diffraction patterns of POD and the POD-LCTs. Various characteristic peaks of POD disappeared in the XRD pattern of the POD-LCTs, which might indicate that the surface structure of the POD-LCTs was made up of carrier materials. This also suggests that POD was encapsulated in the carriers, which masked the crystalline form of POD [33].

#### 2.3.4. FTIR Spectroscopy

FTIR analysis was carried out to study any chemical interaction between the excipients and the drug in the formulations [34,35]. According to the FTIR spectra in Figure 4B, POD showed strong absorption bands at 3500 cm^−1^ and 1768 cm^−1^, which were caused by stretching vibration of –OH and C=O, respectively. The absorption peaks appearing in the range of 3029–2807 cm^−1^ were caused by the stretching vibration of C–H and –CH_2_ in the benzene ring in POD [36]. In the FTIR spectra of the physical mixture and lyophilized POD-LCTs, some characteristic absorbance bands of POD at 1768 cm^−1^ and 3029–2807 cm^−1^ were present, while the absorption peak at 3500 cm^−1^ disappeared, indicating that there might be hydrogen bonding or a dipolar interaction between POD and the excipients [37,38].

#### 2.3.5. Elasticity

The elasticity of the transfersome was evaluated by measuring its ability to pass through pores of a certain size under constant pressure. Elasticity is a very important parameter because it might affect the ability of the transfersome to penetrate through the stratum corneum [13]. The vesicle elasticity of the optimal POD-LCTs was 15.3 ± 0.7 (Table 1), which indicates that Tween-80 in the formulation could soften the vesicle membrane and make the phospholipid bilayer more flexible [39,40]. Therefore, it is easy for the POD-LCTs to change their shape and pass through the stratum corneum.

#### 2.3.6. Stability Study

**Figure 5:**
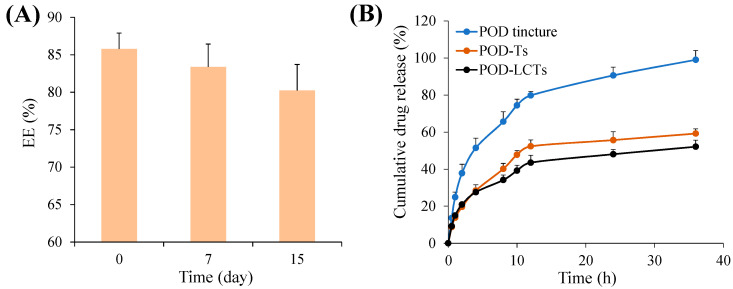
(**A**) EE of POD-LCTs as a function of time over 15 days at 4 °C (*n* = 3). (**B**) The in vitro release of POD from POD tincture, POD-Ts and POD-LCTs in the release medium of physiological saline (*n* = 3).

The stability of the nanoparticles was evaluated at 4 °C for 15 days. It was found that the formulation remained uniform at 4 °C, without any formation of precipitation or aggregation during the storage time. As shown in Figure 5A, the EE value of the POD-LCTs decreased slightly, but there was no statistical difference compared with the value at 0 days. This result indicates that the POD-LCTs had good stability in the short term. It has been reported that the stability of nanoparticles depends strongly on the affinity of the drugs to the hydrophobic core of nanoparticles [41].

### 2.4. In Vitro Release Study

The in vitro drug release of the formulation was studied by using a Franz diffusion cell with a dialysis membrane. Figure 5B shows the in vitro release curve of POD from the POD tincture, POD-Ts and POD-LCTs in a release medium of physiological saline. The cumulative release of POD tincture, POD-Ts and POD-LCTs at 36 h was 99.04 ± 5.00%, 59.24 ± 2.48% and 52.13 ± 3.57%, respectively. Unsurprisingly, the POD-Ts and POD-LCTs exhibited a significantly slower and lower release than the POD tincture (*p* < 0.01). The observed sustained POD release from the transfersomal formulation might be attributed to the tight encapsulation of the drug by the lipid vesicle structure, which acquired a significant advantage over the common tincture dosage forms as a drug repository, thus resulting in sustained release of the encapsulated drug [30,42]. The sustained release behavior of POD from the transfersomes is beneficial for avoiding the drug’s release before the transfersomes penetrate into the skin [43]. In addition, the sustained release behavior could also help the drug to play a long-term role, thus reducing the dose frequency.

The in vitro drug release data of POD-LCTs were fitted into kinetic equations of the zero order, first order, Higuchi and Korsmeyer–Peppas. The fitting results are shown in Table 2. The best fitting model of POD-LCTs was selected according to the correlation coefficient values (R^2^) obtained from the selected models. The results show that the release kinetics of the POD-LCTs was consistent with the Korsmeyer–Peppas model, with an R^2^ value of 0.9660. According to the parameters of the Korsmeyer–Peppas model, it was found that the value of the release exponent “*n*” of the POD-LCTs was 0.3982, indicating that the release mechanism for the POD-LCTs was Fickian diffusion, which describes the release of diffusion-based drugs from a porous polymer skeleton system [44].

### 2.5. In Vitro Skin Permeation and Retention

**Figure 6:**
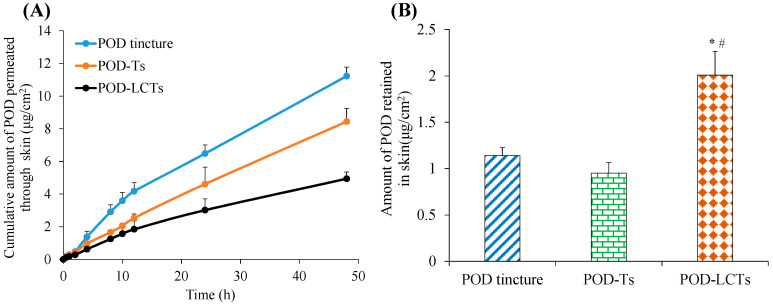
In vitro skin permeation studies of POD tincture, POD-Ts and POD-LCTs in porcine ear skin after 48 h of topical administration. (**A**) The in vitro skin cumulative permeation of POD through the skin. (**B**) POD retention in the skin at 48 h (results are presented as mean ± SD, *n* = 3) * *p* < 0.05 compared with the POD tincture. # *p* < 0.05 compared with the POD-Ts.

In vitro skin permeation studies could provide valuable insights about the in vivo behavior of the formulation [45]. The Franz diffusion cell was used to evaluate the cumulative amount of a drug which permeated through the intact porcine ear skin in this study. As shown in Figure 6A, the cumulative amount of POD which permeated from the POD tincture, POD-Ts and POD-LCTs at the end of 48 h was 11.23 µg/cm^2^, 8.44 µg/cm^2^ and 4.94 µg/cm^2^, respectively. The cumulative amount of drug penetration of the POD-Ts and POD-LCTs was significantly lower than that of the POD tincture (*p* < 0.05). This result indicates that the transfersomes loaded with POD could decrease the amount of POD penetration through the skin. The significant difference in the cumulative amount of drug penetration between the tincture solution and transfersomes might be related to the different methods of penetration promotion. A large amount of ethanol in a POD tincture could promote drug penetration by damaging the stratum corneum of skin [46]. The drug permeation-promoting effect of the transfersomes might be attributed to the following: (1) The surfactant added in the formulation could increase the fluidity of the stratum corneum lipid and increase the space between the keratinocytes [47], (2) since the evaporation of water on the skin surface would cause it to lose some water, the transfersomes would sense the penetration gradient and try to avoid dryness by moving toward the deeper skin along this gradient [48], (3) the transfersomes have ultra-deformable elasticity and flexibility, enabling them to squeeze through the smaller pores between keratinocytes [49], and (4) the small size of the transfersomes could increase the absorbed surface area [50]. On the other hand, the POD-LCTs provided significantly lower drug penetration through the skin compared with the POD-Ts (*p* < 0.05). This might be due to the bioadhesion of sulfhydryl groups on the surface of the POD-LCTs [18]. This result indicates that POD-LCTs might be beneficial for reducing the systemic absorption of the drug, thereby reducing the systemic side effects.

The amount of POD retained in the skin is shown in Figure 6B. Forty-eight hours after administration, the skin retention of the POD tincture, POD-Ts and POD-LCTs was 1.14 µg/cm^2^, 0.95 µg/cm^2^ and 2.01 µg/cm^2^, respectively. There was no significant difference in skin retention between the POD tincture and POD-Ts. However, when compared with the POD tincture and POD-Ts, the skin retention of the POD-LCTs was significantly increased (*p* < 0.05). This might be due to the interaction between sulfhydryl groups on the surface of the POD-LCTs and keratin in the epidermis [21], which promoted the skin accumulation of the drug. In addition, the transfersomes could accumulate in active epidermis and hair follicles, finally prolong drug retention in skin. In conclusion, the POD-LCTs had lower transdermal absorption and higher skin retention compared with the POD-Ts, which were expected to reduce systemic side effects and improve the therapeutic effect of the local diseases.

### 2.6. Fluorescence Distribution Assay

**Figure 7:**
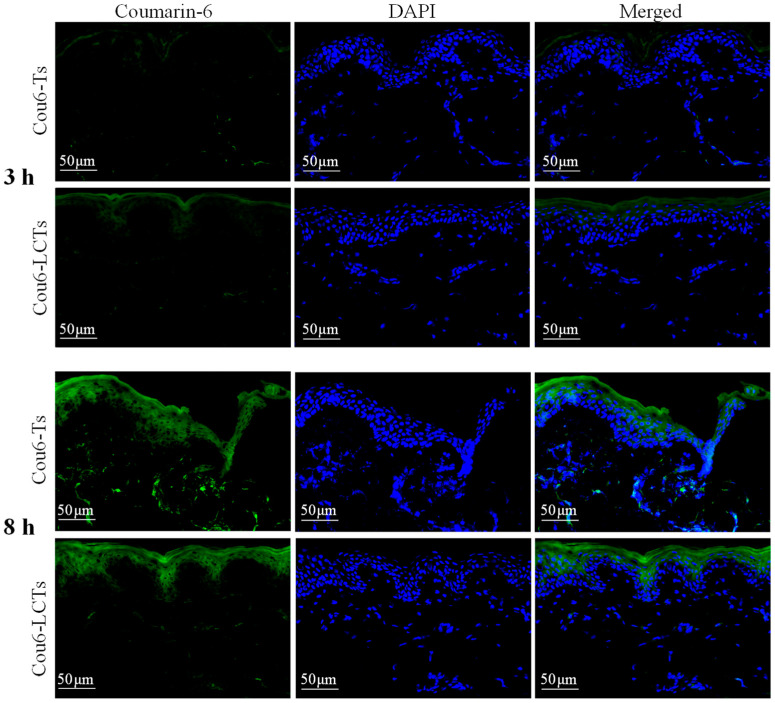
Fluorescence images of vertical section of porcine ear skin applied with Cou6-Ts and Cou6-LCTs at 3 h and 8 h post application.

In order to study the fluorescence distribution of the developed formulation in the skin, a fluorescence imaging experiment was carried out with coumarin-6-loaded formulation. Figure 7 shows the fluorescent images of the vertical sections of porcine ear skin treated with Cou6-Ts and Cou6-LCTs at 3 h and 8 h post administration (the emission in the blue range for the cell nuclei stained by DAPI and green for the coumarin-6-loaded formulation). After the skin was treated with Cou6-Ts and Cou6-LCTs for 3 h, most of the green fluorescent signals were above the blue fluorescent signals, and the green fluorescence signal was weaker in the deeper skin layers. It is well known that the stratum corneum is composed of dead enucleated corneal cells [51]. Therefore, this result shows that most of the formulation was trapped in the stratum corneum at 3 h, and only part of it entered the active epidermis and dermis. This might be due to the fluidity of the stratum corneum surface, which leads to its relatively low penetration resistance [2]. With the increase in the penetration time, the intensity of the green fluorescence in the skin increased significantly. At 8 h after administration, the green fluorescence signal in the skin treated with Cou6-Ts was distributed throughout the epidermis and dermis, indicating that Cou6-Ts could penetrate through the stratum corneum and epidermis into the dermis. However, the green fluorescence signal in the skin treated with Cou6-LCTs was mainly located in the stratum corneum and epidermis, and only a negligible fluorescence signal was observed in the dermis, indicating that Cou6-LCTs could overcome the stratum corneum barrier and specifically gather in the epidermis [52]. These findings confirm that Cou6-LCTs could improve epidermal targeting, which would help improve the epidermal bioavailability of lipophilic drugs and reduce systemic side effects caused by drugs entering into the systemic circulation.

**Figure 8:**
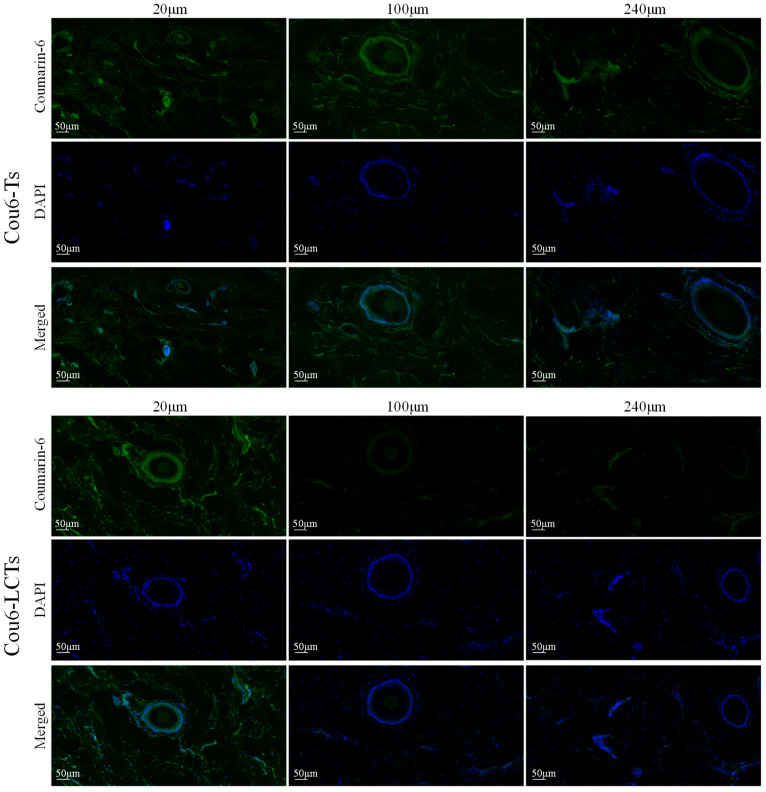
Fluorescence images of horizontal section of porcine ear skin treated with Cou6-Ts and Cou6-LCTs for 8 h.

Figure 8 shows the fluorescence images of the horizontal sections of porcine ear skin at different depths at 8 h after treatment with Cou6-Ts and Cou6-LCTs (the emission in the blue range for the cell nuclei stained by DAPI and the green range for the coumarin-6-loaded formulation). The skin treated with Cou6-Ts showed a significant green fluorescence signal at different depths below the skin’s surface, while the skin treated with Cou6-LCTs only showed a strong green fluorescence signal in the epidermis, and the fluorescence signal gradually decreased with the increase in skin depth. This result further indicates that Cou6-LCTs could be enriched in the stratum corneum and active epidermis, which is an expected position and beneficial for the treatment of genital warts. In addition, green fluorescence signals were observed in the hair follicles and their surrounding areas, which indicates that Cou6-Ts and Cou6-LCTs could also penetrate into the skin through the hair follicles and spread to the tissues around the hair follicles. This might be attributed to the huge opening (10–210 μm) and funnel effect of the hair follicles [53,54].

### 2.7. Histological Examination of the Skin

**Figure 9:**
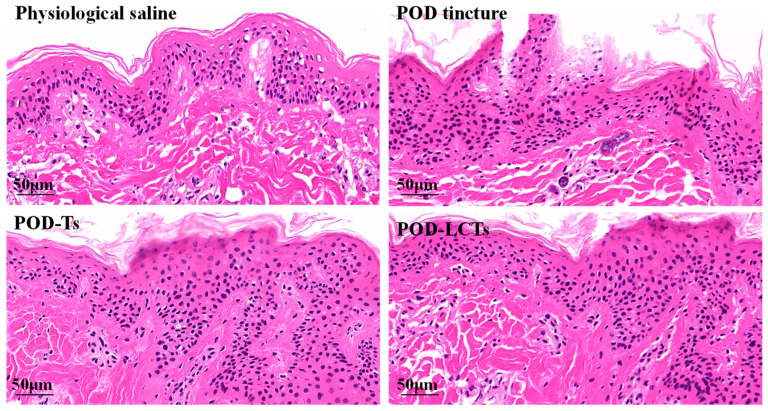
Histological photomicrographs of porcine ear skin stained with HE after incubation with physiological saline, POD tincture, POD-Ts and POD-LCTs.

The effects of the physiological saline, POD tincture, POD-Ts and POD-LCTs on the microstructure of the skin were investigated by HE staining. As shown in Figure 9, the skin treated with physiological saline had a complete structure, and the stratum corneum and epidermis were arranged neatly and connected tightly. A large number of stratum corneum fragments could be seen in the skin treated with the POD tincture, and the epidermal cells were loosely arranged with gaps, indicating that the high concentration of ethanol in the tincture would damage the skin structure, thus allowing the drugs in the POD tincture penetrate into deeper skin layers [55]. The skin treated with POD-Ts and POD-LCTs showed some fragments of detached stratum corneum and a slight increase in the epidermal cell space, which might be due to phospholipids in the formulation blocking the skin’s surface, increasing tissue hydration and changing the highly ordered and dense structure of the skin surface and thus facilitating the drug’s penetration [56,57].

### 2.8. In Vivo Skin Irritation Test

**Figure 10:**
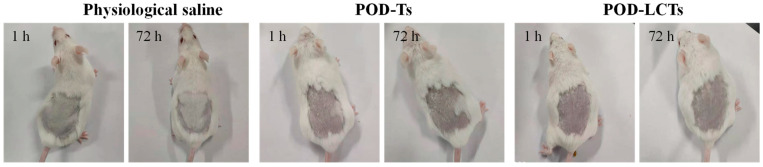
In vivo skin irritation test. Skin appearance of mice observed at 1 h and 72 h after removing the residual formulation of physiological saline, POD-Ts or POD-LCTs (*n* = 6).

The skin irritation of POD-Ts and POD-LCTs was studied in mice with physiological saline as a negative control. The observation results at 1 h and 72 h are shown in Figure 10. The POD-T and POD-LCT formulations did not cause any changes in skin color or morphology, and no sign of erythema or edema was observed throughout the observation period. Therefore, the results indicate that the POD-T and POD-LCT formulations had good biocompatibility and safety, which would help to improve patient acceptance.

## 3. Materials and Methods

### 3.1. Materials

Podophyllotoxin was purchased from Shanxi Haochen Biotechnology Co., Ltd. (Shanxi, China). L-cysteine and coumarin-6 were purchased from Hefei Bomei Biotechnology Co., Ltd. (Hefei, China). Deoxycholic acid was purchased from Guangzhou Renxin Biotechnology Co., Ltd. (Guangzhou, China). Soybean phospholipid was purchased from Hefei BASF Biological Co., Ltd. (Hefei, China), and 1-(3-dimethylaminopropyl)-3-ethylcarbodiimide hydrochloride (EDC.HCL) and N-hydroxysuccinimide (NHS) were purchased from Nanjing Dulai Biotechnology Co., Ltd. (Nanjing, China). Hematoxylin and Eosin Staining Solution were purchased from Fuzhou Feijing Biotechnology Co., Ltd. (Fuzhou, China), while 4′,6-diamidino-2-phenylindole (DAPI) was purchased from Yunke Biotechnology Co., Ltd., (Guangzhou, China), and 4% paraformaldehyde was purchased from Shanghai Rebus Biotechnology Co., Ltd. (Shanghai, China). All other reagents were of an analytical grade.

### 3.2. Synthesis and Characterization of the LC-DCA Conjugate

L-cysteine-deoxycholic acid (LC-DCA) conjugate was synthesized via an amidation reaction between L-cysteine (LC) and deoxycholic acid (DCA). In short, DCA (100 mg) was dissolved in methanol (5 mL) via magnetic stirring for 15 min (30 rpm), and then the DCA solution was stirred for 16 h in the presence of EDC (48 mg) and NHS (35 mg) to activate the carboxyl group. LC (30 mg) was then dissolved in 8 mL of phosphate buffer solution (pH 6.0) and slowly added to the above-mentioned DCA solution. The mixed solution was stirred at 25 °C in the dark for 2 h. The final reaction solution was placed into a dialysis bag (MWCO 300) and dialyzed against an excess amount of deionized water for 48 h to eliminate unreacted constituents. Finally, the solution was filtered through a microporous membrane filter with a pore size of 0.8 μm to remove other impurities, followed by lyophilization at −50 °C for 48 h to obtain LC-DCA powder. The yield of the reactions was 72.6%. The LC-DCA conjugate was stored at 4 °C until further use. The nuclear magnetic resonance (^1^H NMR) spectra and Fourier transform infrared spectroscopy (FTIR) of the LC-DCA were analyzed by using a Nicolet iS50 Fourier transform infrared spectrophotometer (Thermo Fisher Scientific, Waltham, MA, USA) and a 400 MHz apparatus (AVANCE500, Bruker, Germany).

The amount of free sulfhydryl groups in the LC-DCA conjugate was determined through the method of Elman’s regent (DTNB) as described previously [21,58]. First, 2 mg of LC-DCA conjugate was precisely weighed and completely dissolved in 5 mL of 0.5 mol/L PBS (pH = 8.0). Then, 1 mL of Ellman’s reagent (30 mg of DTNB dissolved in 100 mL of 0.5 mol/L PBS) was added to the LC-DCA solution, and the mixture was reacted at 25 °C for 2 h in the dark. Subsequently, the reaction solution was centrifuged at 12,000 r/min for 15 min, and the absorbance of the supernatant was measured at 412 nm by using a microplate reader (PerkinElmer VICTOR Nivo, Turku, Finland). The amount of free sulfhydryl groups was calculated according to the standard curve established by linear regression between a series of cysteine solutions and the corresponding absorbance values.

### 3.3. Preparation of POD-LCTs

POD-loaded L-cysteine modified transfersomes (POD-LCTs) were prepared through a thin membrane dispersion method [45,59]. Components of soybean phospholipid (350 mg), Tween-80 (70 mg) and POD (50 mg) were dissolved in the organic solvent of chloroform. A uniform thin film was formed after the organic solvent was removed under a vacuum for 30 min, with a rotary evaporator at 35 °C. After vacuum drying overnight to remove trace organic solvents, the thin film was then hydrated in LC-DCA aqueous solution at 35 °C for 0.5 h and dispersed by a water bath ultrasonic apparatus in an ice water bath for 8 min. The final formulation of POD-LCTs was obtained by extrusion through the microporous membrane, with a pore size of 0.45 μm, and stored at 2–8 °C for further research. To optimize the amount of LC-DCA in the formulation, a hydrating medium of LC-DCA solution containing 20, 40, 60 and 80 mg of LC-DCA was prepared. The POD-loaded transfersomes (POD-Ts) were prepared with the same method.

### 3.4. Characterization of POD-LCTs

#### 3.4.1. Encapsulation Efficiency (EE)

In order to determine the entrapment efficiency (EE) of the POD-LCTs, the method of ultra-filtered centrifugation was used to separate the non-entrapped POD from transfersomal vesicular system. About 1.0 mL of the formulation was placed in an ultrafiltration centrifuge tube (Millipore, Burlington, MA, USA, MWCO 35 kDa) and subjected to centrifugation at 5000 rpm for 30 min. The filtrate was collected after centrifugation. With necessary dilutions, the drug content in the filtrate was determined with a fluorescence spectrophotometer (F-7000, Hitachi High-Technologies Corporation, Tokyo, Japan) with the excitation wavelength (Ex) and emission wavelength (Em) set at 290 nm and 637 nm, respectively, and the values of the drug content were obtained utilizing the calibration curve of F = 18.185C + 20.589 (R^2^ = 0.9991). The EE% values were calculated according to the following equation:

EE% = (Total drug − non-entrapped drug)/Total drug × 100%

#### 3.4.2. Particle Size and Zeta Potential Measurement

The particle size and zeta potential of the formulation were measured by using a Malvern Zetasizer Nano ZS90 (Malvern instruments, Worcestershire, UK). The prepared formulation was diluted at a 1:200 ratio with deionized water to obtain the optimal kilo counts per second (KCPS) of 50–200 for measurement [60]. The analysis was carried out at 25 °C with an angle of detection of 90°. The average particle size and zeta potential were measured in triplicate.

#### 3.4.3. Morphology Examination with a Scanning Electron Microscope

The morphology of the optimized POD-LCT and lyophilized POD-LCT gel was evaluated using a scanning electron microscope (SEM) (Sigma 500, ZEISS, Jena, Germany) to confirm the formation of POD-LCTs and the integrity of the POD-LCTs after being formulated into gel. For the SEM study of the POD-LCT dispersion, a small drop of a transfersomal suspension diluted 150 times was uniformly coated on the silicon substrate and allowed to dry naturally for 24 h. The POD-LCT gel was freeze-dried under a vacuum at −50 °C for 48 h to fully remove the moisture in the samples for appearance observation. The lyophilized samples were mounted on the aluminum stubs with conductive double-sided adhesive tape. The surface of the samples was gold-coated in a vacuum evaporator. Subsequently, the morphology of the POD-LCT dispersion and lyophilized POD-LCTs was observed with an SEM.

#### 3.4.4. X-ray Diffraction (XRD) and Fourier Transform Infrared Spectroscopy (FTIR)

X-ray diffraction (XRD) and Fourier transform infrared spectroscopy (FTIR) were performed to investigate the crystalline properties of POD in the formulation and possible interaction between POD and the selected excipients [61]. XRD measurements of POD, a physical mixture of POD and the excipients, blank LCTs, and POD-LCTs were conducted using an X-ray difftometer (D8 ADVANCE, Bruker, Germany) under Cu-Kα radiation at a scan rate of 2° per minute in the 2θ range of 10–80°. The above samples were also analyzed by FTIR, and the FTIR spectra of the samples were recorded by a Nicolet iS50 Fourier transform infrared spectrophotometer (Thermo Fisher Scientific, USA) with a frequency of 400–4000 cm^−1^.

#### 3.4.5. Elasticity Measurement

The elasticity of the POD-LCT formulation was measured by the method of microfiltration membrane extrusion according to the previously described method, with minor modifications [62]. Briefly, the POD-LCT formulation was extruded at a constant pressure of 1.0 bar through a microporous membrane filter with a pore size of 0.1 μm for 5 min. The volume of the extruded formulation was measured during the 5 min period, and the particle size was measured by a Malvern Zetasizer Nano ZS90.The elasticity was calculated as follows:

D = J(r_v_/r_p_)^2^

where D is the elasticity of the formulation, J is the volume of the extruded suspension within 5 min, r_v_ is the particle size measured after the formulation extrusion and r_p_ is the pore size of the microporous membrane filter.

#### 3.4.6. Stability of the POD-LCTs

The short-term stability of the drug-loaded POD-LCT nanoparticles was examined at 4 °C. At a series of time points (0, 7 and 15 days), the EE was determined to evaluate the formulation’s stability. In addition, the samples were also qualitatively examined with the naked eye. The presence of precipitation in the formulation indicates instability of the nanoparticles, while a uniform appearance indicates their stability.

### 3.5. In Vitro Release Study

A Franz diffusion cell (RYJ-6B, Shanghai, China) was used to study the in vitro drug release of the developed formulation [63,64]. A dialysis membrane (MWCO 3500 Da) was mounted between the donor and receptor chambers. The diffusion area between the donor chamber and receptor chamber was 2.8 cm^2^, and the volume of the receptor chamber was 6.5 mL. The donor chamber was added with 0.5 mL of formulation (equivalent to 2.5 mg POD), and the receptor chamber was filled with physiological saline as the release medium. The stirring speed was set at 300 rpm, the temperature was maintained at 37 ± 0.5 °C (ensuring that the membrane surface temperature was 32 °C to simulate human skin conditions) throughout the experiment, and the drug release was monitored within 36 h. At predetermined intervals, a 1.0 mL sample was withdrawn from the receptor chamber and immediately replaced with an equal temperature and volume of fresh physiological saline. The drug content was determined with a fluorescence spectrophotometer. The percentage of cumulative drug release was calculated from the concentration of the drug in the release medium and plotted as a function of time. The release curves were fitted by several kinetic equations (zero-order, first-order, Higuchi and Korsmeyer–Peppas models), using nonlinear regression to study the in vitro drug release mechanism [65,66].

### 3.6. In Vitro Skin Permeation and Retention Studies

#### 3.6.1. Preparation of Porcine Ear Skin Samples

Fresh porcine ears were purchased from the slaughter market, and the domestic pigs were inspected by the quarantine department. After slaughter, the porcine ears were immediately removed, the skin was washed with physiological saline and dried with filter paper, the hair on the back of the ears was removed with an electric shaver, and then the ears were cut into strips with a width of 3 cm. The porcine ear strips with intact skin were selected, and the skin was sliced to a thickness of 600 µm with a skin graft knife (Z10100, Shanghai Medical Instrument Co., Ltd., Shanghai, China) and cut into round sheets with a diameter of 30 mm using a round hole punch. The skin samples were wrapped in aluminum foil and stored at −30 °C for use, and the storage time was no more than 3 months.

#### 3.6.2. Skin Permeation and Retention Studies

The skin permeation study was performed according to the relevant OECD guideline “OECD 428 Testing of Chemicals (2004)” (https://wenku.baidu.com/view/bb827d29954bcf84b9d528ea81c758f5f71f2950.html?_wkts_=1690473888015&bdQuery=OECD+428+Testing+of+Chemicals+%282004%29, accessed on 13 April 2004). A vertical Franz diffusion cell (RYJ-6B, Shanghai Huanghai Drug Control Instrument Co., Ltd., Shanghai, China) with an effective diffusion area of 2.8 cm^2^ and a diffusion cell volume of 6.5 mL was used to study the in vitro skin permeation and retention of POD tincture, POD-Ts and POD-LCTs. The experiment of skin permeation was conducted using porcine skin from the back part of the ear. The receptor chamber was filled with degassed physiological saline as the receptor solution. The magnetic stirrer was set at 300 rpm and continuously stirred to maintain the uniformity of the receptor solution. The temperature of the receptor chamber was maintained at 37 °C to keep the skin surface temperature at 32 ± 0.5 °C. The porcine skin sample was mounted between the donor and receptor chamber of the diffusion cell with the stratum corneum layer facing the drug formulation and the dermis facing the receiving solution. Then, 0.5 mL of POD tincture, POD-Ts and POD-LCTs (equivalent to 2.5 mg POD) was added to the donor chamber. The receiving solution (1.0 mL) was withdrawn from the receptor chamber at specified time intervals over a 48 h period and immediately supplemented with an equal volume of freshly preheated physiological saline. A high-performance liquid chromatography (HPLC) system (U-3000, Thermo, Waltham, MA, USA) equipped with a UV detector and reversed-phase WondaSil C18 column (5 μm, 250 × 4.6 mm) was used to analyze the POD concentrations in the receptor chamber at each time point. The mobile phase was composed of methanol-water (60:40, *v*/*v*), the flow rate was set at 1.0 mL/min, and the UV detection wavelength was set at 293 nm. The data were expressed as the amount of POD per area (μg/cm^2^), and the cumulative permeation of each formulation was plotted against time.

At the end of the permeation study, the skin was carefully removed from the diffusion cell, and the residual formulation on the skin surface was fully wiped with deionized water to ensure that no traces of formulation were left on it. The skin permeation area was excised, cut into small pieces, homogenized in 5 mL methanol for 2 min and then subjected to ultrasound at room temperature for 30 min [67]. The obtained samples were centrifuged (3000 rpm) for 10 min, and the clear supernatant was analyzed with HPLC.

### 3.7. Fluorescence Imaging

Coumarin-6 was used as a fluorescent probe to investigate the penetration of the carriers in the porcine ear skin. In vitro skin penetration of the coumarin-6-loaded transfersomes (Cou6-Ts) and coumarin-6-loaded L-cysteine modified transfersomes (Cou6-LCTs) was carried out using a Franze diffusion cell. At 3 h and 8 h, the skin samples were collected and washed with physiological saline to remove the residual formulation on the skin surface. The treated skin was successively cut off and frozen at −80 °C, and then the cross-section perpendicular to the skin surface and the horizontal cross-section parallel to the skin surface were prepared using a freezing slicer (Leica CM 1950, Nussloch, Germany). DAPI was used for nuclear staining of the tissue sections, and fluorescence images were collected using a digital slide scanner (3Dhistech, Budapest, Hungary) to observe the fluorescence distribution in porcine ear skin.

### 3.8. Hematoxylin–Eosin Staining

The porcine ear skin samples were treated with physiological saline, POD tincture, POD-Ts and POD-LCTs for 12 h on Franz diffusion cells, and then the skin samples were collected. The residual formulation on the skin samples was washed with physiological saline. Thereafter, the skin samples were fixed in 4% paraformaldehyde solution and embedded in paraffin. The skin samples were vertically cut into continuous slices with a thickness of 5 μm using a microtome (LEICA RM2235, Nussloch, Germany) and stained with hematoxylin and eosin (HE). Then, images of the paraffin sections were obtained using a digital slide scanner (3Dhistech, Budapest, Hungary) for histological observation.

### 3.9. In Vivo Skin Irritation Test

Female ICR mice (18–22 g) were used as experimental animals for skin irritation tests to evaluate the biocompatibility and safety of the formulated POD-Ts or POD-LCTs. The mice were randomly divided into three groups with six animals in each group. The hair on the dorsal side of the mice was shaved with an electric shaver 24 h before the test, and the skin was observed for abnormalities. The mice were administered with the formulations with an area of approximately 3.0 cm^2^ with the following treatment: Group 1 received physiological saline (negative control), Group 2 received the POD-T formulation (0.5 mL), and Group 3 received the POD-LCT formulation (0.5 mL). The formulation was removed after 4 h of administration on the skin, and the skin was observed at 1, 4, 24, 48 and 72 h for changes in color and morphology, as well as signs of erythema and edema.

### 3.10. Statistical Analysis

All experiments were repeated at least three times, and the results were expressed as the mean ± standard deviation (SD). The differences between three or more independent groups were analyzed with the Student’s *t*-test. In all cases, a *p* value ≤ 0.05 was considered for statistical significance.

## 4. Conclusions

POD-LCTs were prepared through a thin membrane dispersion method for the topical delivery of POD. The in vitro skin permeation and retention studies showed that the POD-LCTs provided significantly lower drug penetration through the porcine ear skin and significantly increased skin retention when compared with POD-Ts (*p* < 0.05). A fluorescence distribution assay showed that the L-cysteine-modified transfersomes were mainly located in the stratum corneum and epidermis, and only a negligible fluorescence signal was observed in the dermis. The POD-LCTs seemed to penetrate into the skin through the pathways of the stratum corneum and hair follicles. The small size and the deformable elasticity and flexibility of POD-LCTs might contribute to the penetration of POD-LCTs into the stratum corneum. The surface modification of L-cysteine might lead to promoting the epidermal delivery of POD-LCTs. This study is helpful for developing a new drug delivery system for POD to improve the topical drug treatment of genital warts and reduce the adverse effects caused by systemic absorption, which is worthy of further study.

## Figures and Tables

**Table 1:** The effect of the amount of LC-DCA on the particle size, potential and EE of POD-LCTs (*n* = 3).

Amount of LC-DCA (mg)	Particle Size (nm)	PDI	Zeta Potential (mV)	EE (%)	Elasticity
0	148.0 ± 56.6	0.13 ± 0.04	−28.6 ± 5.9	84.33 ± 5.16	17.6 ± 0.3
20	165.3 ± 57.9	0.12 ± 0.01	−30.6 ± 5.9	88.16 ± 4.63	16.1 ± 0.5
40	167.8 ± 55.2	0.11 ± 0.04	−30.0 ± 6.4	86.82 ± 2.78	15.9 ± 0.3
60	172.5 ± 67.2	0.14 ± 0.08	−31.3 ± 6.7	84.47 ± 3.84	15.3 ± 0.7
80	192.0 ± 69.9	0.15 ± 0.06	−33.8 ± 5.7	72.34 ± 6.56	15.1 ± 0.5

**Table 2:** Fitting results of cumulative drug release curve of POD-LCTs in vitro.

Kinetic Model	Fitting Equation	Regression Equation	R^2^ Value
Zero order	Mt/M∞ = Kt	Mt/M∞ = 0.0109t	0.7574
First order	Mt/M∞ = 1 × 10^−Kt^	Mt/M∞ = 1 × 10^−0.0169t^	0.8218
Higuchi	Mt/M∞ = Kt^1/2^	Mt/M∞ = 0.0809t^1/2^	0.9235
Korsmeyer–Peppas	Mt/M∞ = Kt^n^	Mt/M∞ = 0.1458t^0.3982^	0.9660

Mt/M∞ is the cumulative drug release percentage at a given moment, t is the release time, and K is the release constant.

## Data Availability

All data generated or analyzed during this study are included in this published article.
